# Catastrophic healthcare expenditure and its inequality for households with hypertension: evidence from the rural areas of Shaanxi Province in China

**DOI:** 10.1186/s12939-016-0506-6

**Published:** 2017-07-01

**Authors:** Yafei Si, Zhongliang Zhou, Min Su, Meng Ma, Yongjian Xu, Jesse Heitner

**Affiliations:** 1 School of Public Policy and Administration, Xi’an Jiaotong University, No. 28 Xianning West Road, Xi’an, Shaanxi 710049 China; 2 Global Health and Population Department, Harvard T.H. Chan School of Public Health, 677 Huntington Avenue, Boston, USA

**Keywords:** Catastrophic health care expenditure, Hypertension, Concentration index, Decomposition, Inequality

## Abstract

**Background:**

China has been undergoing tremendous demographic and epidemiological transitions during the past three decades and increasing burden from non-communicable diseases and an ageing population have presented great health-care challenges for the country. Numerous studies examine catastrophic healthcare expenditures (CHE) worldwide on whole populations rather than specific vulnerable groups. As hypertension and other chronic conditions impose a growing share of the disease burden in China, they will become an increasingly important component of CHE. This study aims to estimate households with hypertension incurring CHE and its income-related inequality in the rural areas of Shaanxi Province.

**Methods:**

Data were obtained from the National Household Health Service Surveys of Shaanxi Province conducted in 2013 and 13104 households were identified for analysis. The households were classified into three types: households with non-chronic diseases, households with hypertension only and households with hypertension plus other chronic diseases. CHE was measured according to the proportion of out-of-pocket health payments to non-food household expenditures and the concentration index was employed to measure the extent of income-related inequality in CHE. A decomposition method based on a probit model was used to decompose the concentration index into its determining components.

**Results:**

The incurring of CHE of households with hypertension is at a disconcerting level compared to households with non-chronic diseases. Households with hypertension only and households with hypertension plus other chronic diseases incurred CHE in 23.48% and 34.01% of cases respectively whereas households with non-chronic diseases incurred CHE in only 13.33%. The concentration index of households with non-chronic diseases is -0.4871. However, the concentration index of households with hypertension only and households with hypertension plus other chronic diseases is -0.4645 and -0.3410 respectively. The majority of observed inequalities in CHE were explained by household economic status and having elder members.

**Conclusions:**

The proportion of households incurring CHE in the rural areas of Shaanxi Province was considerably high in all three types of households and households with hypertension were at a higher risk of incurring CHE. Furthermore, there existed a strong pro-poor inequality of CHE in all three types of households and the results implied more inequality in households with non-chronic diseases compared with two other groups. Our study suggests that more concern needs to be directed toward households with hypertension plus other chronic diseases and households having elder members.

## Background

China has been undergoing tremendous demographic and epidemiological transitions during the past three decades [1]. From 1990 to 2013 China experienced rapid economic growth with life expectancy at birth increasing. However, increasing burden from non-communicable diseases and an ageing population have presented great healthcare challenges for China. Rising income inequality has also increased policy attention on the challenges of health inequalities [2]. Concerns have been raised that health inequalities are rising [3].

**Fig. 1:**
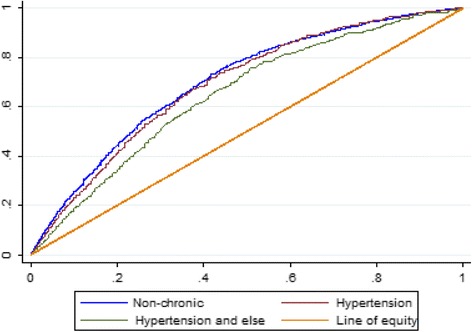
Concentration curves of facing catastrophic health care expenditure (CHE) in rural Shaanxi, China

Chronic diseases occur more frequently in elderly people and often carry high economic burdens; hence, the prevalence of chronic diseases is rising with the rapidly aging population, which overburdens households with health expenditures and increases societal costs. Unlike most western countries that have had this transition at a slower pace, China has experienced this shift only in a few decades [4, 5], which has consequently caused a rapid increase in chronic disease burden. The prevalence of individual chronic diseases ranged from 3.0% for tumor to 76.4% for hypertension, and each disease was often accompanied with three or more other chronic diseases [6]. Chronic diseases were responsible for 75% of all deaths in 2013, and their financial burden accounted for about 70% of the total economic disease burden [7]. Several studies suggested that hypertension is the main determinant of high cerebrovascular disease levels in China [8, 9] and the 5th National Health Service Surveys (NHSS) of Shaanxi Province indicated that the prevalence of hypertension in rural areas of Shaanxi province was as high as 12%, ranking first among the rural residents of all diseases with heavy economic burdens.

Three social health insurance schemes have been implemented in China now: the Urban Employee Basic Medical Insurance (UEBMI) designed for the employed urban residents, the Urban Resident Basic Medical Insurance (URBMI) designed for urban residents without formal employment, and the New Rural Cooperative Medical Insurance (NRCMI) designed for rural residents [10, 11]. However, evidence indicates that households covered by the NRCMI had similar levels of catastrophic health expenditure and medical impoverishment as those without health insurance [12] and the coverage of NRCMI showed no financial protection for households with chronic diseases [13].

The fundamental goal of a health system is to not only improve population health outcomes but also protect households from illness-associated financial catastrophe [14]. However, millions of people are still prevented from obtaining needed healthcare owing to economic status [15]. Catastrophic health care expenditure (CHE) is a general term used to describe all types of health expenditures that pose a threat to the financial capacity of a household to maintain its subsistence needs [16–19]. Generally, two thresholds are widely used to define CHE: ① out-of-pocket healthcare payments (OOP) that comprise ≥10% of total household expenditures [20–23]; and ② out-of-pocket healthcare payments that comprise ≥40% of nonfood household expenditures [24–27]. By deducting food expenses, the latter indicator can partly avoid measurement deviation that results from ignoring poor households which cannot afford to meet catastrophic payments [28]. Despite the fact that “average” catastrophic health spending could be reduced with policy interventions, inequalities in CHE will not simply be eliminated and inevitably exist across households due to geographic and economic factors [29].

Households with members suffering from chronic diseases have a greater chance of experiencing catastrophic health expenditure than households without, which is consistent in both developed countries and developing countries. Evidence from the Republic of Korea showed that although Korea has greatly expanded its health insurance coverage, financial protection against CHE remains a concern and roughly 3.5% of households experienced CHE while 7.3% households with hypertension experienced CHE [30]. Meanwhile, there is significant evidence of substantial cost burden placed by NCDs on patients living in low and middle income countries, with most of it being heavily concentrated among low socioeconomic status groups [31]. NCDs pose a heavy financial burden on many affected households, and poor households are the most financially affected when they seek care [32]. Unfortunately, the literature on the social, financial and economic consequences of NCDs in developing countries has not kept pace with the epidemiological evidence [33].

China is facing high disease burden, with OOP that payments remain relatively high, and the overall incidence of catastrophic health expenditure is about 13% [34, 35]. Rapid increasing demand for affordable access to health services has also been accompanied by a progressive shift in burden from infectious diseases to non-communicable diseases. In view of the demographic trends in China, this shift is highly likely to continue with many health system implications. Many studies indicate that households with members who have chronic conditions face higher financial risks than other households, and there is distributive income inequality in terms of CHE incidence and intensity [36–38]. Several Chinese studies report that in rural China households with members who have chronic conditions have higher financial risks than other households [23–26, 39], and their incidence of CHE is almost 1.5 times greater than the average level of whole population [20].

Numerous studies examine catastrophic healthcare expenditures (CHE) worldwide on the whole populations rather than specific vulnerable groups [7, 12, 13, 29]. An insurance scheme that ignores the disease profile and health expenditure pattern of the population can have only limited effectiveness in protecting the population from medical impoverishment [40]. However, few studies have been conducted to analyze specific diseases’ impact on health system performance in China and studying the effect of specific chronic diseases upon catastrophic health expenditure may aid in strengthening disease-dependent benefit coverage. As hypertension and other non-communicable chronic conditions impose a growing share of the disease burden in China, they will become an increasingly important component of CHE. This study aims to estimate households with hypertension incurring CHE and its income-related inequality in the rural areas of Shaanxi Province.

Based on the cross-sectional data from the 5th National Health Services Survey in Shaanxi province, this study provided evidence on the extent, relevant factors and inequality of catastrophic health expenditure in households with hypertension and other chronic diseases in rural China. In view of the prominence of hypertension and other chronic disease as a cause of death and the huge gradients across the country, our findings may contribute to improving and adjusting related health policy, thereby further relieving the economic burden of hypertension and other chronic diseases.

## Methods

### Data source

Shaanxi Province is located in the northwest of China and 48.7% of residents live in rural areas. Data were obtained from the 5th National Health Service Surveys (NHSS) of Shaanxi conducted in 2013. NHSS is a nationally representative survey of China organized and directed by the National Health and Family Planning Commission of China every 5 year [29]. A four-stage, stratified, random sampling method was adopted in 2013 to achieve representation of the whole population. Finally, 32 counties (districts) in Shaanxi Province, 160 townships, 320 villages (communities) and then 20700 households were identified [41]. We focus on the rural component of the survey in which 13200 households in rural areas were identified. After data cleaning (i.e. excluding households with logic error answers and/or with key variables missing), 13104 households were identified for analysis.

The household questionnaire included general information on socio-economic and demographic characteristics of households, insurance characteristics, self-reported illness and injury, and outpatient and inpatient health service utilization of household members. Nine questions were employed in the questionnaire to measure annual household expenditure on food, accommodation, transportation, and OOP health expenditure and so on. The recall period for household expenditure was one year prior to the survey [42]. An adult who was fully aware of the household economic information was eligible to respond in this section.

Three questions were employed to identify whether the interviewees had chronic diseases. ➀ Were you diagnosed with hypertension by doctors in the last six months? ➁ Were you diagnosed with diabetes by doctors in the last six months?➂. Were you diagnosed with other chronic diseases by doctors in the last six months? The households were classified into three types: households with non-chronic diseases, households with hypertension only and households with hypertension plus other chronic diseases. In the end, 11433 households with non-chronic diseases (87.26%), 1082 households with hypertension only (8.26%) and 588 households with hypertension plus other chronic diseases are identified (4.49%).

### Measuring CHE incidence

We measured CHE using the indicators reported by Wagstaff et al. [34, 43]. We used non-food household expenditure instead of total household expenditure as the denominator in order to calculate CHE, and thereby partly avoid measurement deviations that are often ignored in poor households. Following Xu et al. [44], we considered health care expenditure ‘catastrophic’ if it was equal to or higher than 40% of the household capacity to pay, and defined a dummy variable to capture this. Capacity to pay was defined as household consumption expenditure minus basic subsistence needs adjusted for household size. Xu et al. [44] have reported the methodology in detail [44]. Following Wang et al. [6], overshoot and mean positive overshoot were employed to identify CHE intensity. Overshoot measures the extent by which an average OOP health expenditure crosses the given catastrophic threshold of the entire sample, while mean positive overshoot indicates the extent by which the average OOP health expenditure of a household exceeded the given threshold [7].

### Methods to measure CHE inequality

Concentration index (CI) was employed to measure the extent of socioeconomic inequality in CHE. It is defined as twice the area between the concentration curve and the line of equality [42, 45]. The concentration index lies in [−1, 1] [46], and its positive value indicates that a variable is more concentrated among the rich, and vice versa. The larger the absolute value of concentration index indicates the greater the inequality in CHE [47]. The formula for computing the concentration index is:

1 $$ \mathrm{C}=\frac{2}{\mu} c o v\left({y}_i,{R}_i\right) $$

Where C is concentration index, *y*_*i*_ is CHE indicator, μ is the mean of CHE indicator and *R*_*i*_ is the fractional rank of household in the economic status distribution. Given that the economic status was measured by annual household expenditure, poor households spending catastrophic expenditure on health services increased their “capacity to pay” (CTP) and total expenditure, thus these households were categorized into a higher economic status in analyses. Therefore, an additional economic status was generated utilizing annual household expenditure minus OOP to define the per-capita expenditure.

### Decomposition methods

Inequality can be further explained by decomposing the concentration index into its determining components [47]. Decomposition methods can enable researchers to quantify each determinant’s specific contribution to measure income-related inequality while controlling for other determinants.

As CHE is a dummy variable, a probit model is employed to decompose the inequality of CHE. In order to be consistent with the method of decomposing the concentration index, independent variables in the regression model are classified into three groups: economic status, need variables and other control variables (Table 1) [7, 29, 30, 48]. As the probit model is a nonlinear model, the linear approximation to the nonlinear model is made by estimating the partial effects evaluated at the covariate means [47]. The regression model is given by Eq. 2:

**Table 1:** Description of independent variables

Need variables		Control variables	
variables	description	variables	description
Illness	1 = Having members of illness in last two weeks, 0^a^ otherwise	Economic status	household consumption expenditure
Inpatient	1 = having members of inpatient in last half year, 0 ^a^otherwise	Marriage status	0^a^ = Not married,1 = married,2 = else marriage of house head
Elderly Members	1 = having members over 60 years old,0^a^ otherwise	Supplementary medical insurance	1 = Having members covered by supplementary medical insurance, 0^a^ otherwise
Score	natural logarithm of house head’s self-report health score	Household scale	0^a^ if residents < =2 1 if 2 < residents < =4 2 if residents > 5
		Migrants scale	0^a^ if migrants = 0 1 if 0 < migrants < =2 2 if migrants > =3
		Time	natural logarithm of minutes to go to the nearest medical institution
		Gender	1 if female house head, 0^a^ otherwise
		Drinking ratio	Drinking counts/residents
		Education level	0^a^ if illiteracy, 1 if elementary, 2 if middle school, 3 if high school, 4 if university of house head

*Note:* Dummy were created for category variables in regression model, ^a^ indicate reference group

2 $$ \mathrm{y}={\alpha}^m+{\displaystyle \sum_j}{\beta}_j^m{x}_j+{\displaystyle \sum_k}{\gamma}_k^m{Z}_k+\varepsilon $$

where y is CHE indicator, *β*_*m*_^*j*^ and *γ*_*k*_^*m*^ are partial effects (i.e. dy/dx_j_ and dy/dz_k_) of each variable and evaluated at sample means; ε is the error term. The decomposition of the concentration index C could be specified as:

3 $$ \mathrm{C}={\displaystyle {\sum}_j}\left(\raisebox{1ex}{${\beta}_j^m\overline{x_j}$}\!\left/ \!\raisebox{-1ex}{$\mu $}\right.\right){C}_j+{\displaystyle {\sum}_k}\left(\raisebox{1ex}{${\gamma}_k^m\overline{Zk}$}\!\left/ \!\raisebox{-1ex}{$\mu $}\right.\right){C}_k+\raisebox{1ex}{$ G{C}_{\varepsilon}$}\!\left/ \!\raisebox{-1ex}{$\mu $}\right. $$

Where μ is the mean of the dependent variable, C_j_ and C_k_ are the concentration indices for x_j_ and z_k_, $\overline{x_j}$ and $\overline{z_k}$ are the means of x_j_ and z_k_. The first term on the right side of Eq. 3 denotes the contribution of need variables to inequality, the second denotes the contributions of control variables, the last term is the generalized concentration index of ε.

The horizontal inequity (HI) index of CHE is computed by subtracting the contribution of need variables from the concentration index of CHE, which is used to measure the equity of CHE. Need is an elusive concept that has been given a variety of interpretations in relation to the definition of equity in health care delivery [49–51]. Healthcare need reflects the residents’ need for health service utilization, which was measured by gender, age, self-reported health status and illness in the last two weeks following the literature [51]. Economic status is measured by household consumption expenditure per year. Consumption expenditure is used rather than income because income is more likely to be misreported and consumption expenditure is a better proxy for resources available [52, 53]. We use per-capita consumption expenditure rather than household consumption expenditure to rule out variation in household size in measuring economic status. Theoretically, households in lower economic status will have higher proportion of CHE [7, 29, 30]. The contribution of economic status is equal to the product of the elasticity of incurring CHE and the inequality of households’ consumption expenditure. Here for CHE, need variables include illness in the last two weeks, inpatients in the last half year, self-reported health score of house head and having elderly members. We expect that low self-reported health score of house head, having illness in the last two weeks, having inpatients in the last half year and having elderly members will lead to a higher risk of incurring CHE. Apart from need variables, other control variables in the model include household consumption expenditure, marital status, education level and gender of household head, drinking ratio in the household, supplementary medical insurance, migrants scale, household population and distance to the nearest health facility. In 2013, the self-reported health score was measured by scores ranging from 0 to 100. As the basic social insurances in China had almost achieved universal coverage, we use supplementary medical insurance as a proxy of healthcare coverage. According to related researches [7, 29], we hypothesized that households with hypertension only and households with hypertension plus other chronic diseases would have a higher proportion incurring CHE; there would exist pro-poor inequality in all three types of households and more inequality of CHE would exist in households with hypertension only and households with hypertension plus other chronic diseases. All analyses were performed in Stata version 11.0 (StataCorp LP, College Station, Texas, USA).

## Results

Given the self-reported chronic diseases in the 5th National Household Health Service Surveys of Shaanxi Province, the households were classified into three types: households with non-chronic diseases, households with hypertension only and households with hypertension plus other chronic diseases. In total, 11433 households with non-chronic diseases (87.26%), 1082 households with hypertension only (8.26%) and 588 households with hypertension and other chronic diseases (4.49%) were identified.

Table 2 shows summary statistics for independent variables. In households with hypertension only, 23.73% heads of household were female, 22.37% were illiterate and 5.82% of them were single. The percentage of households having elderly members was 36.97% in households with hypertension only, and this proportion was 13.54% for households with non-chronic diseases and 42.35% for households with hypertension plus other chronic diseases. In households with hypertension only, 66.73% of households had members with illness in last two weeks and 14.60% of them had members with inpatient utilization in last half year. Only 3.74% of households with hypertension plus other chronic diseases, 5.74% of households with non-chronic diseases and 7.02% of households with hypertension only, were covered by supplementary medical insurance. Univariate ANOVAs showed that between the three groups, there existed statistically significant differences in numerous variables.

**Table 2:** Summary statistics for independent variables

	Non-chro		Hyp		Hyp and else		
	Mean	S.D.	Mean	S.D.	Mean	S.D.	*p*-value
Food expenditure	5541	5028	5107	4873	4830	4731	0.018
Consumption expenditure	21014	16701	19223	17356	19574	16273	<0.001
Per capita consumption expenditure	4818	4153	4321	4004	4185	3607	<0.001
Score	4.37	0.18	4.32	0.20	4.23	0.26	<0.001
Time	2.32	0.95	2.30	0.97	2.40	0.99	0.090
Drinking ratio	0.10	0.25	0.07	0.21	0.04	0.18	<0.001
Gender	0.1895	0.3919	0.2373	0.4256	0.3066	0.4615	<0.001
Inpatient	0.0726	0.2595	0.146	0.3533	0.3129	0.4641	<0.001
Illness	0.121	0.3262	0.6673	0.4714	0.8044	0.397	<0.001
Elderly members	0.1354	0.3422	0.3697	0.4829	0.4235	0.4945	<0.001
Supplementary medical insurance	0.0574	0.2326	0.0702	0.2557	0.0374	0.1899	0.022
House head’s education							
Illiteracy	0.1467	0.3538	0.2237	0.4169	0.2313	0.4220	<0.001
Elementary	0.3130	0.4637	0.3244	0.4684	0.3367	0.4730	0.404
Middle school	0.4083	0.4915	0.3290	0.4701	0.3129	0.4641	<0.001
High school	0.0892	0.2851	0.0804	0.2720	0.0901	0.2866	0.638
University	0.0119	0.1084	0.0083	0.0909	0.0051	0.0713	0.195
Marriage status							
Single	0.0712	0.2572	0.0582	0.2343	0.0629	0.2430	0.021
Married	0.8314	0.3744	0.7800	0.4144	0.7670	0.4231	<0.01
Else-marriage	0.0974	0.2966	0.1617	0.3684	0.1701	0.3760	<0.001
Household scale							
Residents < =2	0.5241	0.4994	0.6562	0.4752	0.7041	0.4568	<0.001
Residents 2-4	0.3810	0.4856	0.2551	0.4361	0.2211	0.4153	<0.001
Residents > =5	0.0950	0.2932	0.0887	0.2845	0.0748	0.2633	0.223
Migrants scale							
No migrants	0.4930	0.5000	0.5055	0.5002	0.4932	0.5004	<0.001
Migrants 1-2	0.2340	0.4234	0.1959	0.3971	0.2024	0.4021	<0.001
Migrants > =3	0.2730	0.4455	0.2985	0.4578	0.3044	0.4606	0.223

*Note*: Univariate ANOVAs was employed for continuous variables and chi-2 test was used for category variables

### Catastrophic health care expenditure

Table 3 displays the average household OOP health expenditure, average household CTP, and the proportion of households with CHE in household. Mean OOP healthcare payments by households with non-chronic diseases was RMB 2952, which accounts for 16.99% of average non-food household expenditures (or capacity to pay). In contrast, mean OOP healthcare expenditure by households with hypertension only was RMB 3330, which comprised 21.03% of average non-food household expenditures. Average OOP healthcare expenditure by households with hypertension plus others chronic diseases was RMB 4709, which comprised 28.78% of average non-food household expenditures. The poorest households had the highest proportion of CHE occurrence compared to other quintiles. Households with hypertension plus other chronic diseases have the highest proportion of CHE occurrence compared to households with non-chronic diseases and households with hypertension only in the same quantile. Not only the poorest households but also the richest households had a high proportion of CHE incurrence in households with hypertension and other chronic diseases. The overall proportion of households incurring CHE dropped from 34.01% in households with hypertension plus other chronic diseases to 23.48% in households with hypertension only and households with non-chronic diseases had the lowest proportion of CHE occurrence at 13.33%, with a statistically significant difference at the level of α =0.01 (Pearson chi2(2) = 251.3314, P < 0.0001). Overshoot (2.19%, 4.08% and 6.87%) and mean positive overshoot (16.45%, 17.36% and 20.20%) have the same trends in three groups.

**Table 3:** Summary statistics for OOP, CTP and CHE

		Poorest	Poorer	Middle	Richer	Richest	All
OOP	Non-chronic	2287.30	2550.10	2854.72	3057.06	4013.26	2952
	Hypertension	2378.66	3352.36	2980.83	3096.64	4856.84	3330
	Hypertension and else	3019.58	3420.42	4664.66	5920.34	6547.86	4709
	p-value	0.031	<0.001	<0.001	<0.001	<0.001	<0.001
CTP	Non-chronic	5471.32	9215.98	13688.45	20350.67	38227.57	17380
	Hypertension	4635.55	8450.70	11313.45	17624.12	37316.36	15835
	Hypertension and else	4915.02	8260.34	13035.33	19446.03	36329.90	16361
	p-value	0.905	0.187	0.004	0.006	0.548	0.004
CHE (%)	Non-chronic	35.37	15.75	8.30	4.39	2.76	13.33
	Hypertension	57.14	32.41	16.59	7.37	3.72	23.48
	Hypertension and else	71.19	38.66	30.17	18.64	11.11	34.01
	p-value	<0.001	<0.001	<0.001	<0.001	<0.001	<0.001

*Note*: Univariate ANOVAs was employed for continuous variables and chi-2 test was used for category variables

### Determinants of CHE

Table 4 shows the results of estimated partial effects and corresponding standard deviations (S.D.) in the probit regression models. As expected, most variables increased the risk of incurring CHE. These results indicate a significant negative correlation between CHE incidence and household economic status and household scale, which means that CHE are more likely to occur in low-income smaller households. This effect was estimated to be greater in households with hypertension only and households with hypertension and other chronic diseases in comparison with households with non-chronic diseases. Health status of the head of household was negatively associated with CHE incidence in all three groups. In other words, health status of the head of household improved as CHE risk decreased. This effect was estimated to be smaller in households with hypertension only and households with hypertension and other chronic diseases in comparison with households with non-chronic diseases. Having elderly household members significantly increased the incidence of CHE in households with non-chronic diseases, while time required to go to the nearest medical institution was negatively associated with CHE incidence in households with non-chronic diseases. Interestingly, neither affected the CHE risk in households with hypertension only and households with hypertension and other chronic diseases. Supplementary medical insurance did not significantly affect the incidence of CHE in all three groups. CHE risk was significantly higher when household members went to hospitals for inpatient services, and this effect was estimated to be smaller in households with hypertension only and households with hypertension and other chronic diseases in comparison with households with non-chronic diseases. However, among households with hypertension only, CHE was not statistically or significantly affected when household members had illness. CHE incidence significantly increased only when household members had illness in households with non-chronic diseases and households with hypertension and other chronic diseases. All three groups had a large and significant constant in this regression. It implies that even accounting for all the variables included, “something” unmeasured (if only randomness in the universe) is contributing to large amounts of inequality.

**Table 4:** Partial effects of socio-economic associates with CHE

	Non-chro		Hyp only		Hyp plus others	
	dy/dx	S.D.	dy/dx	S.D.	dy/dx	S.D.
Economic status	-0.403***	-0.0144	-0.464***	-0.0424	-0.410***	-0.0523
elementary	-0.0898*	-0.0483	-0.0787	-0.131	-0.139	-0.166
Middle school	-0.161***	-0.0514	-0.163	-0.146	-0.261	-0.181
High school	-0.0756	-0.0747	-0.344	-0.244	-0.723**	-0.289
University	0.213	-0.194	-0.263	-0.724	0.546	-0.88
Score	-0.998***	-0.0865	-0.759***	-0.261	-0.714***	-0.238
Time	-0.0363**	-0.0179	0.00247	-0.0515	-0.0824	-0.0666
Residents 2-4	-0.199***	-0.0393	-0.297**	-0.125	-0.490***	-0.168
Residents>=5	-0.152**	-0.061	-0.902***	-0.229	-0.454*	-0.251
Migrants 1-2	-0.123***	-0.0436	-0.219	-0.135	-0.319*	-0.173
Migrants>=3	-0.254***	-0.0432	-0.293**	-0.121	-0.258*	-0.146
Married	0.175**	-0.085	0.167	-0.331	-0.0698	-0.322
Else-marriage	0.0463	-0.0963	0.24	-0.347	-0.239	-0.363
Gender	-0.0319	-0.0475	-0.208	-0.133	0.0495	-0.158
Illness	0.217***	-0.0482	0.175	-0.108	0.407**	-0.171
Inpatients	0.799***	-0.0544	0.522***	-0.132	0.696***	-0.133
Elderly members	0.160***	-0.0458	0.152	-0.109	0.194	-0.13
Drinking ratio	-0.096	-0.0702	-0.22	-0.258	0.139	-0.366
Supplementary medical insurance	0.0784	-0.0801	0.153	-0.206	0.315	-0.323
constant	4.338***	-0.387	3.603***	-1.151	3.681***	-1.106
N	11063		1044		573	

* *p* < 0.1, ** *p* < 0.05, *** *p* < 0.01

### Income-related inequality of CHE

The concentration indices of CHE of households with non-chronic diseases, households with hypertension only and households with hypertension and other chronic diseases are negative, which demonstrates that the poor are more likely to incur CHE than the rich in rural areas of Shaanxi province. However, as the need of household healthcare has not been taken into account, inequality is not equivalent to inequity. The concentration indices of facing CHE were -0.4871 (95% Confidence Interval:-0.5203 to -0.4537), -0.4645 (95% Confidence Interval:-0.5348 to -0.3922) and -0.3410 (95% Confidence Interval:-0.4082 to -0.2715) for households with non-chronic diseases, households with hypertension only and households with hypertension and other chronic diseases, respectively. Testing concentration curve dominance indicated statistically significant dominance of the curve of households with hypertension and other chronic diseases against the curve of households with non-chronic diseases but no significant change in inequality in CHE between households with hypertension only and households with hypertension and other chronic diseases (Fig. 1).

### Decomposition of inequality of CHE

After decomposing the concentration indices of CHE, the income-related inequalities were decomposed into the contributions of different variables (as show in Table 5). The absolute value of contribution signifies the extent to which inequality can be attributed to this variable. The positive value of contribution means the variable contributes to pro-poor inequality, that is, the poorer households had higher probabilities of facing CHE than the rich, and vice versa.

**Table 5:** Decomposition of inequality in CHE

	Elasticity			Concentration index(CI)			Contribution to CI			Contribution to CI(%)		
	non-chro	hyp	else	non-chro	hyp	else	non-chro	hyp	else	non-chro	hyp	else
Economic status	-1.4414	-1.3395	-1.1123	0.2626	0.2897	0.2929	-0.3785	-0.3880	-0.3257	77.71	83.54	95.52
Elementary	-0.0324	-0.0263	-0.0458	-0.0939	-0.0639	-0.0550	0.0030	0.0017	0.0025	-0.63	-0.36	-0.74
Middle school	-0.0762	-0.0546	-0.0788	0.1072	0.1005	0.1111	-0.0082	-0.0055	-0.0088	1.68	1.18	2.57
High school	-0.0076	-0.0248	-0.0521	0.1747	0.2621	0.2498	-0.0013	-0.0065	-0.0130	0.27	1.40	3.82
University	0.0034	-0.0020	0.0031	0.6132	0.4249	0.8132	0.0021	-0.0008	0.0025	-0.43	0.18	-0.74
Score	-5.1398	-3.4231	-3.0026	0.0037	0.0049	0.0036	-0.0188	-0.0167	-0.0108	3.86	3.60	3.17
Time	-0.0993	0.0059	-0.1968	-0.0327	-0.0229	-0.0450	0.0032	-0.0001	0.0089	-0.67	0.03	-2.60
Residents 2-4	-0.0869	-0.0735	-0.0983	0.0747	0.1123	0.1399	-0.0065	-0.0083	-0.0138	1.33	1.78	4.03
Residents>=5	-0.0157	-0.0557	-0.0296	-0.0124	0.0913	0.1462	0.0002	-0.0051	-0.0043	-0.04	1.09	1.27
Migrants 1-2	-0.0325	-0.0418	-0.0603	0.0219	0.0273	0.0384	-0.0007	-0.0011	-0.0023	0.15	0.25	0.68
Migrants>=3	-0.0754	-0.0857	-0.0757	-0.0246	0.0457	0.0651	0.0019	-0.0039	-0.0049	-0.38	0.84	1.44
Married	0.1572	0.1292	-0.0538	0.0278	0.0463	0.0676	0.0044	0.0060	-0.0036	-0.90	-1.29	1.07
Else-marriage	0.0055	0.0436	-0.0384	-0.2071	-0.1929	-0.2881	-0.0011	-0.0084	0.0111	0.23	1.81	-3.24
Gender	-0.0070	-0.0487	0.0152	0.0779	-0.0082	0.0415	-0.0005	0.0004	0.0006	0.11	-0.09	-0.18
Illness	0.0345	0.1184	0.2999	-0.0221	0.0005	0.0105	-0.0008	0.0001	0.0032	0.16	-0.01	-0.92
Inpatients	0.1029	0.0936	0.2280	-0.0193	-0.0121	-0.0344	-0.0020	-0.0011	-0.0078	0.41	0.24	2.30
Elderly members	0.0277	0.0596	0.0824	-0.3047	-0.1578	-0.1420	-0.0084	-0.0094	-0.0117	1.73	2.03	3.43
Drinking ratio	-0.0119	-0.0153	0.0060	0.0171	0.1602	0.2636	-0.0002	-0.0025	0.0016	0.04	0.53	-0.46
Supplementary medical insurance	0.0056	0.0120	0.0126	0.2185	0.0821	0.2170	0.0012	0.0010	0.0027	-0.25	-0.21	-0.80

*Note*: There are 11063 observations when decomposing the inequality of CHE in households with non-chronic diseases, 1044 observations when decomposing the inequality of CHE in households with hypertension only and 573 observations when decomposing the inequality of CHE in households with hypertension and other chronic diseases

Tables 5 shows elasticity, the CI, contribution to CI and relative contributions of each related factor of CHE inequality(contribution to CI %) in households with non-chronic diseases, households with hypertension only and households with hypertension and other chronic diseases. A positive contribution to socioeconomic inequality means that the relevant variable increases inequality, and vice versa.

As shown in Table 5, the majority of the observed inequalities in incidence in households with non-chronic diseases can be attributed to economic status (77.71%), middle school(1.68%), score(3.86%), residents 2-4 (1.33%) and having elderly members (1.73%). The total contribution percentage is 84.39%, which means that 15.61% of the negative contribution to inequality in incidence is explained in the error term of the regression. In households with hypertension only, the major contribution to inequality is associated with economic status (83.54%), with middle school (1.18%), high school (1.40%), score (3.60%), residents 2–4 (1.78%), more than 5 residents (1.09%), married (-1.29%), else marriage (1.81%) and having elderly members (2.03%) also contributing. The total contribution percentage is 96.52%, which means that 3.46% of the negative contribution to inequality in incidence is explained in the error term of the regression. The last column of Table 6 shows that the main contribution to inequality in incurring CHE in households with hypertension and other chronic diseases is associated with economic status (95.52%), middle school (2.57%), high school (3.82%), score (3.17%), time (-2.60%), residents 2-4 (4.03%), more than 5 residents (1.27%), more than 3 migrants (1.44%), married (1.07%), else marriage (-3.24%), inpatient (2.30%) and having elderly members (3.43%). The total contribution percentage is 109.62%, which means that 9.62% of the positive contribution to inequality in incidence is explained in the error term of the regression.

**Table 6:** Concentration index, need variables’ contribution and horizontal inequity index in CHE

	Non-chronic	Hypertension	Hypertension and else
CI	-0.4871	-0.4645	-0.3410
Need	-0.0300	-0.0272	-0.0272
HI	-0.4571	-0.4373	-0.3138

As the variables were divided into four groups: economic status, need variables, control variables and the residual term. The contribution of each variable-group was generated by adding up contributions of variables within each group. The sum of CHE would be zero if CHE were equal across economic status and the need variables would be the only ones to indicate differences if there were perfect equity. Among these contributions, economic status made the greatest contribution to the inequality of incurring CHE in all three groups and all of the contributions were negative, indicating that most of the pro-poor inequalities are accounted for by economic status. The contributions of need variables on the inequality of probability of incurring CHE are negative all three groups, meaning that poorer household have greater need, while the contributions on the inequality of error term in households with hypertension and other chronic diseases was positive, suggesting that the wealthy households have higher probabilities of incurring CHE.

The horizontal inequity indices in CHE were calculated by using the method of decomposition of the concentration index. As shown in Table 6, all of the horizontal inequity indices of CHE of households with non-chronic diseases, households with hypertension only and households with hypertension and other chronic diseases are negative, which indicates that CHE inequities exist for rural households in Shaanxi province, and that the poor is more likely to incur CHE than the rich when they have otherwise the same socioeconomic statuses (pro-poor inequity). Compared to the inequity of CHE in households with non-chronic diseases, the inequity of CHE in households with hypertension only and households with hypertension and other chronic diseases were lower and the inequity of CHE in households with hypertension and other chronic diseases was lowest. As the need variables contribute less than control variables, the horizontal inequity index of CHE in households with non-chronic diseases, households with hypertension only and households with hypertension and other chronic diseases have the same trend in rural areas of Shaanxi province.

## Discussion

This study uses the cross-sectional data from the 5th National Health Services Survey in Shaanxi Province to study the incidence, intensity and inequality of CHE for households with non-chronic diseases, households with hypertension only and households with hypertension plus other chronic diseases. To the best of our knowledge, it is the first to analyze the extent, relevant factors of, and inequality of CHE in households with hypertension.

Indirect expenditure for seeking health services, such as transportation, accommodation and lost earnings due to illness, were not included in OOP and this conservative estimation method may lead to underestimating the financial consequences of household health expenditures [29]. Although such a conservative method was employed to measure CHE, the proportion of households incurring CHE in Shaanxi Province was still considerably high in all three groups, as demonstrated in earlier studies [7, 12, 13, 29, 30]. Our results implied higher probability of incurring CHE in households with hypertension only and households with hypertension plus other chronic diseases. We observed that significant difference occurred in the proportion of facing CHE in households with non-chronic diseases, households with hypertension only and households with hypertension plus other chronic diseases.

Households with non-chronic diseases, households with hypertension only and households with hypertension plus other chronic diseases have differences in many aspects. Generally, households with hypertension only and households with hypertension plus other chronic diseases are more vulnerable than households with non-chronic diseases to incur CHE. This study emphasized several key factors as determinants of catastrophic health care expenditure and most were similarly reported in related studies, such as economic status, household size and so on [7, 29]. As we expected, a lower economic status played an important role in increasing the risk of incurring CHE in all three groups. A small household with more illness in last two weeks, having elderly members and with an illiterate head of households, had higher risk of incurring CHE. Therefore, policy interventions aimed at reducing the probability of household incurring CHE should primarily consider the needs of these vulnerable households. Specifically, large household size protects against CHE, which is more common in rural areas. We use supplementary medical insurance as a proxy of healthcare coverage. The finding that healthcare coverage did not significantly affect CHE is similar to some existing literatures [12, 13, 40]. This may imply that the social health insurance programs in China actually may not have reduced the risk of catastrophic spending and relieved financial burden in rural areas and even that increasing the financing level could have limited effect in reducing CHE in rural Shaanxi province. The weak performance of social health insurance in financial protection maybe caused by the high prevalence of chronic diseases among the elderly population and the corresponding medical expenditure pattern in policy design [40]. Increasing compensation for hypertension and other chronic diseases should be a practicable way to improve the effectiveness and sustainability of the health insurance system in China.

Given that the economic status was measured by annual household expenditure, poor households spending catastrophic expenditure on health services increased their CTP and total expenditure, thus these households were categorized into a higher economic status in analyses. Therefore, we generated a new economic status with annual household expenditure minus OOP and then get the per-capital expenditure. By doing so, we found that the CI was bigger than that calculated by other researchers as expected [7, 29], which implies the underestimation of CHE in earlier studies. Furthermore, the CI of households with non-chronic diseases is smaller than that of households with hypertension only and households with hypertension plus other chronic diseases, which is the reverse of our hypothesis. One plausible explanation is that hypertension and other chronic diseases have higher incidence in the well-off and therefore reduce the inequality of incurring CHE.

After decomposing the inequality of incurring CHE, we find that economic status made the greatest pro-poor contribution to the inequality of incurring CHE in all three groups. In other words, rising incomes in rural areas of China increased income differentials in incurring CHE. As previously noted, the contribution of economic status is equal to the product of the elasticity of incurring CHE and the inequality of households’ consumption expenditure. Theoretically, the contribution of each determinant to the change of the concentration index of incurring CHE can be attributed to an interaction of changes, which includes the change of this determinant, the change of the determinant’ concentration index, and the change of partial effects of the determinant on incurring CHE. Because we cannot change the elasticity of incurring CHE, the only effective way to increase the equality of incurring CHE is to reduce the inequality of households’ consumption expenditure. As Table 6 demonstrates, the consumption expenditure concentration index was larger in households with hypertension only and households with hypertension plus other chronic diseases than in households with non-chronic diseases (0.2897, 0.2929 and 0.2626 respectively), which is not consistent with the fact that the inequalities in the probability of CHE in three groups. This can be explained by a smaller elasticity indices of incurring CHE in households with hypertension only and households with hypertension plus other chronic diseases. The contributions of need variables to the inequality of incurring CHE are similar in three groups so the horizontal inequity indices are consistent with the fact that the inequalities in the probability of CHE in three groups. Therefore, the results imply more inequity in households with non-chronic diseases than the other two types of households. Furthermore, the results also show that residual variables contributed extensively to the increase in pro-poor inequality, suggesting that there remains a good deal of unexplained variation in changes in inequity beyond the variables examined in this analysis.

There are several limitations to this study which should be noted. First, although we have tried our best to identify hypertension and other chronic diseases’ contribution to CHE and its inequalities. OOP of households with hypertension only and of households with hypertension plus other chronic diseases is health expenditure of all members in the household, not just that caused by hypertension. Methods trying to capture direct contribution of hypertension and other chronic diseases are still needed. Second, the use of self-reported measures of chronic disease may substantially underestimate prevalence in low-income and middle-income country settings, especially in groups with lower socioeconomic status [54]. Third, Self-reported health status can be considerably affected by residents’ health consciousness level and health knowledge level [48]. Last but not least, indirect expenditure for seeking health services were not included in OOP and this conservative estimation may lead to underestimating the financial consequences of household health expenditures [29]. Therefore, the incidence and inequality of incurring CHE may be underestimated in this paper.

## Conclusion

The proportion of households incurring CHE in the rural areas of Shaanxi Province was considerably high in all three types of households and more seriously households with hypertension were at a higher risk of incurring CHE. It seems that currently the NRCMI has limited effect to prevent catastrophic health expenditure in households with hypertension. Furthermore, there existed a strong pro-poor inequality of CHE in all three types of households but the results imply more inequality in households with non-chronic diseases compared with two other groups. Our study suggests that more concern needs to be directed toward households with hypertension plus other chronic diseases and households having elder members. More importantly, policy makers should focus on improving financial protection and relieving the economic burden of households with hypertension plus other chronic diseases, thereby reducing CHE and alleviating CHE inequality among households with hypertension in rural China. Increasing compensation for hypertension and other chronic diseases should be a practicable way to improve the effectiveness and sustainability of health insurance system in China.

## Abbreviations

CHE: Catastrophic Healthcare Expenditure
CI: Concentration Index
CTP: Capacity to Pay
HI: Horizontal inequity
NHSS: National health service surveys
NRCMI: New rural cooperative medical insurance
OOP: Out-of-Pocket healthcare payments

## References

[1] Zhou M, Wang H, Zhu J (2016). Cause-specific mortality for 240 causes in China during 1990–2013: a systematic subnational analysis for the Global Burden of Disease Study 2013. Lancet.

[2] Yang DT (1999). Urban-biased policies and rising income inequality in China. Am Econ Rev.

[3] Tang S, Meng Q, Chen L (2008). Tackling the challenges to health equity in China. Lancet.

[4] Yang G, Kong L, Zhao W (2008). Emergence of chronic non-communicable diseases in China. Lancet.

[5] Yang G, Wang Y, Zeng Y (2013). Rapid health transition in China, 1990–2010: findings from the Global Burden of Disease Study 2010. Lancet.

[6] Wang R, Yan Z, Liang Y (2015). Prevalence and patterns of chronic disease pairs and multimorbidity among older Chinese adults living in a rural area. PLoS ONE.

[7] Wang Z, Li X, Chen M (2015). Catastrophic health expenditures and its inequality in elderly households with chronic disease patients in China. Int J Equity Health.

[8] He J, Klag MJ, Wu Z (1995). Stroke in the People's Republic of China. II. Meta-analysis of hypertension and risk of stroke. Stroke.

[9] Yong H, Foody JA, Linong J (2013). A systematic literature review of risk factors for stroke in China. Cardiol Rev.

[10] Lin W, Liu GG, Chen G (2009). The urban resident basic medical insurance: a landmark reform towards universal coverage in China. Health Econ.

[11] Tang S, Tao J, Bekedam H (2012). Controlling cost escalation of healthcare: making universal health coverage sustainable in China. BMC Public Health.

[12] Li Y, Wu Q, Liu C (2014). Catastrophic health expenditure and rural household impoverishment in China: what role does the new cooperative health insurance scheme play?. PloS ONE.

[13] Jing S, Yin A, Shi L (2013). Whether New Cooperative Medical Schemes reduce the economic burden of chronic disease in rural China. PLoS ONE.

[14] Xu K. Distribution of health payments and catastrophic expenditures methodology. Geneva: Department of Health System Financing, World Health Organization; 2005.

[15] Hajizadeh M, Nghiem HS (2011). Out-of-pocket expenditures for hospital care in Iran: who is at risk of incurring catastrophic payments?. Int J Health Care Finance Econ.

[16] WP SE, Aizuddin AN, Zainuddin Z, Manaf MRA, Aljunid S (2012). Catastrophic health expenditure and its influencing factors in Malaysia. BMC Health Serv Res.

[17] Berki SE (1986). A look at catastrophic medical expenses and the poor. Health Affair.

[18] Wyszewianski L (1986). Families with catastrophic health care expenditures. Health Serv Res.

[19] Wagstaff A, Van Doorslaer E (2002). Catastrophe and impoverishment in paying for health care: with applications to Vietnam 1993–1998. Health Econ.

[20] O’Donnell O, van Doorslaer E, Rannan-Eliya A, Somanathan CG (2005). Explaining the incidence of catastrophic payments for health care: comparative evidence from Asia. EQUITAP Working Paper No.5.

[21] Galarraga O, Sosa-Rubi SG, Galarraga O, Salinas-Rodriguez A, Sesma-Vazquez S (2010). Health insurance for the poor: impact on catastrophic and out-of-pocket health expenditures in Mexico. Health Econ.

[22] Bredenkamp C, Mendola M, Gragnolati M (2011). Catastrophic and impoverishing effects of health expenditure: new evidence from the western Balkans. Health Policy Plan.

[23] Limwattananon S, Tangcharoensathien V, Prakongsai P (2007). Catastrophic and poverty impacts of health payments: results from national household surveys in Thailand. Bull World Health Organ.

[24] Xu K, Evans DB, Kawabata K (2003). Household catastrophic health expenditure: a multicountry analysis. Lancet.

[25] Kavosi Z, Rashidian A, Pourreza A (2012). Inequality in household catastrophic health care expenditure in a low-income society of Iran. Health Policy Plan.

[26] Onwujekwe O, Hanson K, Uzochukwu B (2012). Examining inequities in incidence of catastrophic health expenditures on different healthcare services and health facilities in Nigeria. PLoS ONE.

[27] Van Minh H, Phuong N, Saksena P, James CD, Xu K (2013). Financial burden of household out-of-pocket health expenditure in Viet Nam: findings from the national living standard survey 2002–2010. Soc Sci Med.

[28] Wagstaff A, van Doorslaer E (2003). Catastrophic and impoverishment in paying for health care: with application to Vietnam 1993–98. Health Econ.

[29] Xu Y, Gao J, Zhou Z (2015). Measurement and explanation of socioeconomic inequality in catastrophic health care expenditure: evidence from the rural areas of Shaanxi Province. BMC Health Serv Res.

[30] Choi JW, Choi JW, Kim JH (2015). Association between chronic disease and catastrophic health expenditure in Korea. BMC Health Serv Res.

[31] Goryakin Y, Suhrcke M (2014). The prevalence and determinants of catastrophic health expenditures attributable to non-communicable diseases in low-and middle-income countries: a methodological commentary. Int J Equity Health.

[32] Lee JT, Hamid F, Pati S (2015). Impact of noncommunicable disease multimorbidity on healthcare utilisation and out-of-pocket expenditures in middle-income countries: cross sectional analysis. PLoS ONE.

[33] Kankeu HT, Saksena P, Xu K (2013). The financial burden from non-communicable diseases in low-and middle-income countries: a literature review. Health Res Policy Syst.

[34] Wagstaff A, Lindelow M (2008). Can insurance increase financial risk? The curious case of health insurance in China. J Health Econ.

[35] Wu QH, Li Y, Xu L, Hao YH (2012). Effect of health insurance on reduction of catastrophic health expenditure in China. Chin J Health Policy.

[36] Onoka CA, Hanson K, Onwujekwe O, Uzochukwu B (2011). Examining catastrophic health expenditures at variable thresholds using household consumption expenditure diaries. Trop Med Int Health.

[37] Flores G, Krishnakumar J, O’Donnell O, van Doorslaer E (2008). Coping with healthcare costs: implications for the measurement of catastrophic expenditures and poverty. Health Econ.

[38] Yardim MS, Cilingiroglu N, Yardim N (2010). Catastrophic health expenditure and impoverishment in Turkey. Health Policy.

[39] Chen YY, Yin AT, Zhao WJ, Chen YY, Yin AT, Zhao WJ (2012). Research on the association of rural residents disease economic risk and disastrous health spending. Health Econ Res.

[40] Yip W, Hsiao WC (2009). Non-evidence-based policy: how effective is China's new cooperative medical scheme in reducing medical impoverishment?. Soc Sci Med.

[41] Zhou Z, Gao J (2011). Study of catastrophic health expenditure in China’s basic health insurance. Health Med.

[42] Doorslaer E, Koolman X (2004). Explaining the differences in income‐related health inequalities across European countries. Health Econ.

[43] O'Donnell O A, Wagstaff A. Analyzing health equity using household survey data: a guide to techniques and their implementation. World Bank Publications. 2008

[44] Xu K. Distribution of health payments and catastrophic expenditures methodology. 2005. http://apps.who.int/iris/handle/10665/69030.

[45] Kakwani N, Wagstaff A, Van Doorslaer E (1997). Socioeconomic inequalities in health: measurement, computation, and statistical inference. J Econ.

[46] Wagstaff A (2005). The bounds of the concentration index when the variable of interest is binary, with an application to immunization inequality. Health Econ.

[47] Wagstaff A, Van Doorslaer E. Measuring and testing for inequity in the delivery of health care. J Hum Resour. 2000;716–733.

[48] Zhou Z, Gao J, Fox A (2011). Measuring the equity of inpatient utilization in Chinese rural areas. BMC Health Serv Res.

[49] Culyer AJ (1995). Need: the idea won't do—but we still need it. Soc Sci Med.

[50] Culyer AJ, Wagstaff A (1993). Equity and equality in health and health care. J Health Econ.

[51] Zhou Z, Su Y, Gao J (2011). New estimates of elasticity of demand for healthcare in rural China. Health Policy.

[52] Wang H, Zhang L, Hsiao W (2006). Ill health and its potential influence on household consumptions in rural China. Health Policy.

[53] Meyer B D, Sullivan J X. Measuring the well-being of the poor using income and consumption. No. w9760. Natl Bur Econ Res. 2003. doi:10.3386/w9760.

[54] Vellakkal S, Millett C, Basu S, et al. Are estimates of socioeconomic inequalities in chronic disease artefactually narrowed by self-reported measures of prevalence in low-income and middle-income countries? Findings from the WHO-SAGE survey. J Epidemiol Community Health. 2014; jech-2014-204621 10.1136/jech-2014-204621 PMC4345525 25550454

